# Influence of Beijing Winter Olympic Games Construction on Vegetation Coverage around Zhangjiakou Competition Zone

**DOI:** 10.3390/ijerph182312777

**Published:** 2021-12-03

**Authors:** Yuan Zhang, Zhongqi Xu, Jiabing Wu

**Affiliations:** 1 Key Laboratory of Forest Ecology and Management, Institute of Applied Ecology, Chinese Academy of Sciences, Shenyang 110016, China; zhangyuan18@mails.ucas.ac.cn; 2 College of Resources and Environment, University of Chinese Academy of Sciences, Beijing 100049, China; 3 College of Foresty, Hebei Agricultural University, Baoding 071001, China; Xzq7110@163.com

**Keywords:** Winter Olympic Games, vegetation coverage changes, environmental disturbance

## Abstract

There is a rising concern that Olympic venue construction may affect the surrounding environment. The construction of Winter Olympic venues and competition zones is more likely to degrade the surrounding natural environment than the summer counterpart, considering the prominent land use change and extensive vegetation disturbance during the construction of ski trails in mountainous areas. Scientific assessment of the impact of this Winter Olympic Games construction on the surrounding ecological environment can be of significance for the construction of a Green Olympics. At this stage, the main framework of venue and competition construction in Zhangjiakou for the Beijing Winter Olympic Games is essentially completed, so we assessed the vegetation coverage change conditions based on the Normalized Difference Vegetation Index (NDVI) and the Enhanced Vegetation Index (EVI) from 2000 to 2020. Our results show that the construction of venues, roads, and other facilities for the 2022 Olympic Games led to a remarkable change in land use, but the impacts on vegetation coverage were negligible in the surrounding area. Due to the intensive reforestation activities since the year that Beijing won the race to host the Winter Olympics, vegetation coverage continued to increase in the Zhangjiakou area, even in the core area of Winter Olympic Games construction zones. This study provides support to the belief in hosting a Green Olympics.

## 1. Introduction

Since the first Winter Olympic Games were held in 1924, 23 Winter Olympic Games have been successfully held in 12 countries. In the early days, due to the limited number of participants, the Olympic Games were usually hosted by small cities, whereas after the 1970s, the scale of the competition became larger, and the host cities were mostly medium-sized and large cities [1]. Hosting the Olympic Games can improve the transportation of the host city [2] and enhance the possibility of sustainable development [3]. However, on the other hand, the environmental costs of hosting a sporting mega-event can be high [4]. The Winter Olympic Games are mostly held in mountain resorts, and are thus close to the natural ecosystem, which leads them to encounter strong opposition from environmental organizations [5]. There were some negative concerns about vegetation destruction and serious ecological degradation in the competition zones [6]. For instance, public referenda held in Denver turned down the International Olympic Committee’s offer to host the 1976 Winter Games due to concerns of environmental destruction [7,8]. The construction of the Jeongseon Alpine Centre in Mount Gariwang for the PyeongChang Winter Olympics was also intensely argued against because more than 500-year-old forests on the mountain had been destroyed to create Olympic ski slopes [9]. Therefore, the ecological impact that is caused by the construction of the Winter Olympic Games is worthy of attention.

Large-scale vegetation coverage changes can be monitored with vegetation indices of remote sensing. The vegetation indices are a series of band combinations composed of visible and near-infrared bands. Among a variety of vegetation indices that calculated from different spectral bands, the Normalized Difference Vegetation Index (NDVI) and Enhanced Vegetation Index (EVI) are the most common monitoring indices of vegetation coverage in terrestrial ecosystems [10]. The performances of these two indices differ. EVI was developed from NDVI, which solves the problem of NDVI index saturation in areas with high vegetation coverage, and reduces the influence of land background and aerosols [11,12], while the disadvantages of EVI are that it is too sensitive to topographic effects [13,14].

Since Beijing won the bid to host the 2022 Winter Olympic Games in 2015, the construction of competition zones has quickly begun. As one of the three competition zones for the 2022 Beijing Winter Olympic Games, Zhangjiakou will host snowboarding, freestyle skiing, cross-country skiing, ski jumping, Nordic combined, and biathlon competitions. By the end of 2020, the construction of the main framework of the Zhangjiakou competition zone had essentially been completed. The construction of eight venues and auxiliary projects in the Chongli District of the Zhangjiakou zone was fully completed in the first half of 2021. In the remaining time before the opening of the Winter Olympic Games, there will mostly be no more extensive disturbance to vegetation.

The Beijing Organizing Committee for the 2022 Winter Olympic Games has released its official “Beijing 2022 Olympic and Paralympic Winter Games Sustainability Plan”, and has promised to hold a Winter Olympic Games with a positive environmental impact. Now is the time to evaluate the impact of large-scale Winter Olympic venue and facility construction on the natural environment. The Beijing Winter Olympics involves three competition zones: Beijing, Yanqing, and Zhangjiakou. Here, we focused our research only on the Zhangjiakou competition zone, considering that the overall construction intensity of the Yanqing competition zone was much smaller than that of Zhangjiakou, while the Beijing competition zone construction was mostly based on the venues of the 2008 Beijing Summer Olympic Games, which hardly involves any destruction of vegetation. In this article, we assess the vegetation coverage changes in the Zhangjiakou competition zone based on remote sensing vegetation indices from 2000 to 2020, especially during the time period from 2015 to 2020, to reveal the impact of Winter Olympic Games construction on the natural environment in the surrounding region. At the same time, from the perspective of vegetation restoration and protection, we also discuss the significance of positive measures regarding the implementation of the Sustainability Plan, including afforestation in the whole host city region and the establishment of the Olympic Forest Park to transplant tens of thousands of big trees from the construction area. This study provides a reference for the ecological assessment of large-scale project construction, and also provides support to the belief of hosting a Green Olympics in the future.

## 2. Materials and Methods

### 2.1. Study Area

**Figure 1:**
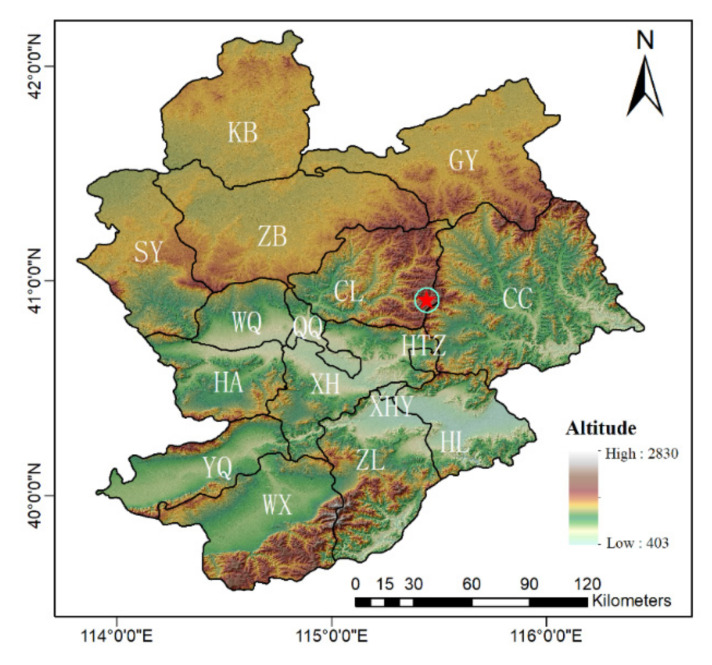
Digital Elevation Model (DEM) shading map of Zhangjiakou City, Hebei Province, China. The light blue words denote the abbreviation of the districts or counties (see Table 1 for full names). The venue zone is indicated by a red five-pointed star. The pentagram part with red circle is the core area of Winter Olympic Games construction zone, and its center is the venue zone, a 6 km radius including Thaiwoo resort, Wanlong resort, Genting resort, Taizicheng, and surrounding villages. All the venues and trails are within the circle, within a radius of six kilometers.

Our research area is located in the area of Zhangjiakou City, Hebei Province, China, which is one of the three competition zones of Beijing Winter Olympic Games. The whole region has a temperate continental monsoon climate, with precipitation between 300 and ~500 mm, and an average altitude of 1148 m. There are 16 districts or counties in Zhangjiakou (Figure 1). All the venues of Zhangjiakou competition Zone of Winter Olympic Games are located in Chongli district.

### 2.2. Moderate Resolution Imaging Spectroradiometer (MODIS) Vegetation Indices (MOD13Q1) Data Products

MOD13Q1 is one of the generated products of Terra and Aqua satellites, which were launched by National Aeronautics and Space Administration (NASA). This product, with spatial resolution of 250 m, was downloaded from the NASA website (https://ladsweb.nascom.nasa.gov/search/ (accessed on 10 October 2020)). The data product contained Normalized Difference Vegetation Index (NDVI), Enhanced Vegetation Index (EVI), two quality layers, reflectance bands (red, near-infrared, blue, and mid-infrared), and four observation layers [15]. We selected 84 images obtained in July and August from 2000 to 2020. Maximum Value Composite [16] was used to generate the vegetation map for each year. These images were used to assess vegetation conditions of all areas.

### 2.3. Landsat-8 Operational Land Imager (OLI) Data Products

Landsat-8 OLI is one of the two sensors of Landsat-8 satellite, which was launched in 2013. This product, with spatial resolution of 30 m, is a Level-2 Data Product, and was downloaded from the United States Geological Survey (USGS) website (https://earthexplorer.usgs.gov/ (accessed on 10 October 2020)). The data product was corrected with radiation and geometry. We selected eight images obatined in late August in 2013 and 2020. The cloud content of almost all images was less than 10%, which ensured that there was no cloud in the core area. These images were used to assess vegetation conditions of the core areas in Zhnagjiakou competition zone.

### 2.4. Algorithms for Calculating and Mapping

Two vegetation indices of MOD13Q1 were used to estimate the vegetation coverage change trend of all study areas. The tendency of vegetation was calculated by least-square method [17].

(1) $$\theta =\frac{\sum_{i}^{n}\left(x_{i}-\bar{x}\right)\left(y_{i}-\bar{y}\right)}{\sum_{i}^{n}{\left(x_{i}-\bar{x}\right)}^{2}}$$

In the formula, θ, *i*, $x_{i}$, and $y_{i}$ represent the slope of the trend, the ordinal number, year, and corresponding vegetation index.

The core area with a radius of six kilometers only occupied a small part in relation to the whole study area, so this part was also analyzed with Landsat-8 data, considering that its spatial resolution is finer. Through the comparison of the images of Landsat-8 from late August from 2013 to 2020, the construction of Winter Olympic Games was not earlier than 2015. Meanwhile, in 2015, Beijing was awarded the right to host the Winter Olympics. Therefore, 2015 can be considered as the starting year for Beijing Winter Olympics construction.

Considering the high altitude and the low vegetation coverage, as well as the highly undulating terrain, both the vegetation indices of NDVI and EVI were taken into account in this study. Their calculation formulas are as follows:

(2) $$\mathrm{NDVI}=\frac{\mathrm{NIR}-\mathrm{RED}}{\mathrm{NIR}+\mathrm{RED}}$$

(3) $$\mathrm{EVI}=2.5*\frac{\mathrm{NIR}-\mathrm{RED}}{\mathrm{NIR}+6*\mathrm{RED}-7.5*\mathrm{BULE}+1}$$

The average change in tendency of vegetation was calculated by Equation (4) (since only the first and last two of the six images from 2015 to 2020 were qualified in the core area, we did not use Equation (1) to calculate). The µ value was assessed through subtraction, with θ calculated by Equation (1) (from 2000 to 2014) to judge vegetation change conditions between 2015 and 2020. In order to better understand the vegetation change trend before and after the construction of Winter Olympic Games, a different trend map of the changes was created.

(4) $$\text{µ}=\frac{X_{2020}-X_{2015}}{2020-2015}$$

In the formula, µ represents the average change tendency of vegetation, and X_2015_ and X_2020_ represent the vegetation indices corresponding to the year.

## 3. Results

### 3.1. The Vegetation Coverage Changes in the Core Area of Winter Olympic Games Zones

During the first 15 years (2000–2014), the vegetation increased with a θ value of 0.0041 (calculated by NDVI, and the corresponding EVI is 0.0036. If not specifically stated below, the number in parentheses after the NDVI value is the corresponding EVI) and *p* < 0.05 (0.05). While during the last 5 years (2015 and 2020), the performances of the two vegetation indices were different, the vegetation increased with the µ value of 0.0019 (−0.0050), which is clearly lower than the former stage. This indicates that the vegetation in the core area was actually influenced during the construction of the Winter Olympic competition zones, considering that no remarkable climatic changes were observed during recent years in the study area [18].

The difference of the two tendency indicators (θ and µ) that reflect the change trend of different times can show the influence of human activities on vegetation under the condition of vegetation coverage change. The mean of differences is −0.0022 (−0.0086), which indicates that in some way vegetation was destroyed, or at least, the natural growth of local vegetation was affected.

**Figure 2:**
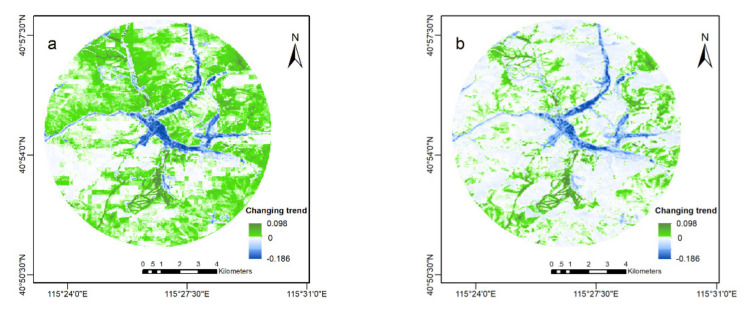

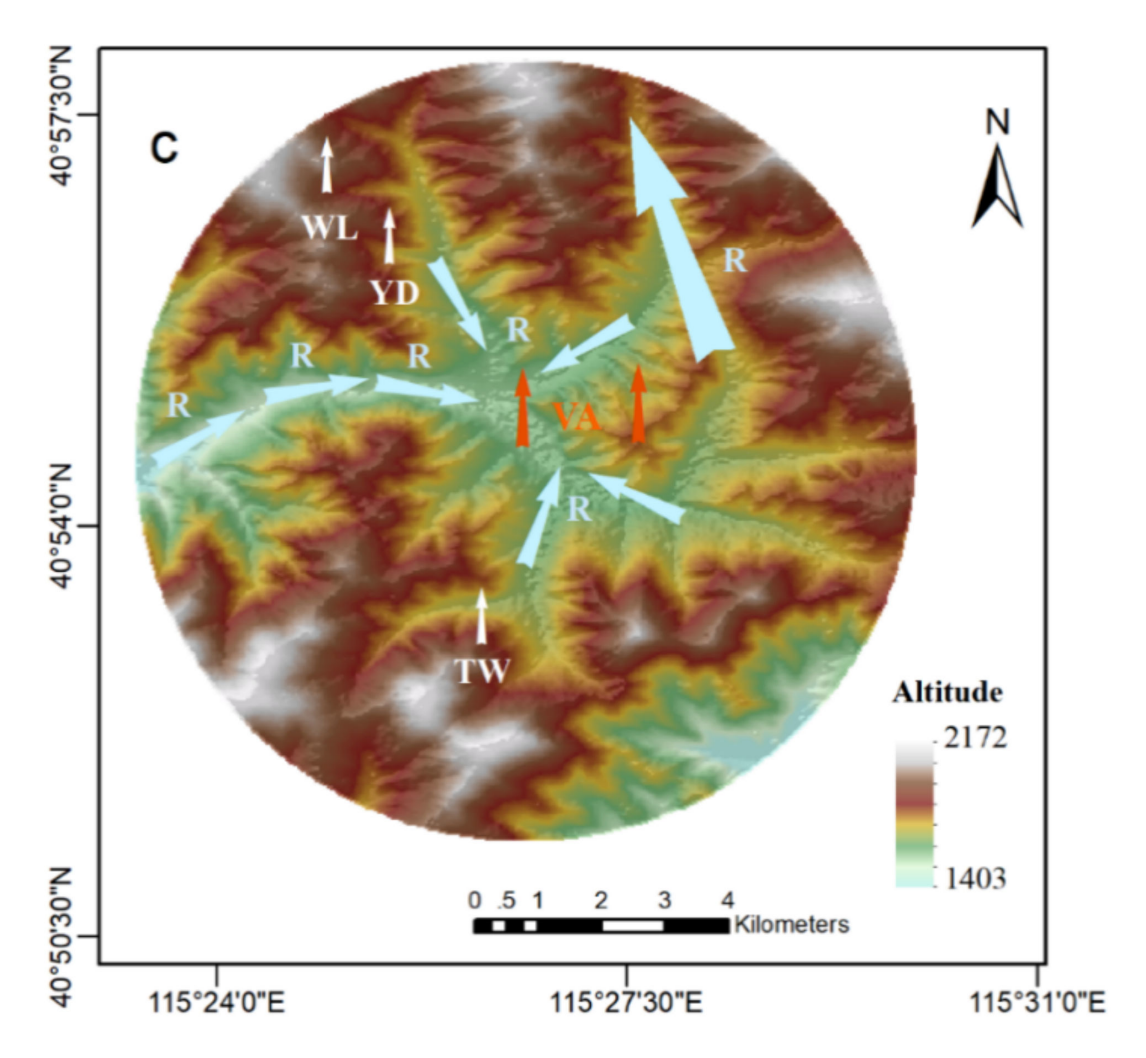
Increasing trend of vegetation in the surrounding areas of Chongli competition zone of Beijing Winter Olympic Games. (**a**,**b**) show the difference of vegetation change trend between 2000 and 2014 and between 2015 and 2020 (i.e., µ_2015–2020_–θ_2000–2014_) of Normalized Difference Vegetation Index (NDVI) and Enhanced Vegetation Index (EVI), respectively. The color is directly proportional to vegetation coverage changes. The deeper the green, the greater increase of vegetation coverage; the opposite for blue; the white means no change. (**c**) shows the Digital Elevation Model shading map; the white arrow shows the resorts (WL, YD, and TW: Wanlong, Genting, and Thaiwoo resort, respectively), and the corresponding light red and light blue are the venue area (VA) and road (R), respectively.

The core area is located in Chongli District, and the three main ski resorts were constructed in the core area. As it can be seen from Figure 2, the vegetation cover decreased remarkably in a concentrated and continuous way, which was very different from the stable increase in the surrounding areas, and it highly overlapped with the construction area of Winter Olympic venues in the Zhangjiakou competition zone. This indicates that the construction of venues, roads, and other facilities for the Olympic Games led to significant land-use change. From north to south are the Wanlong and Genting resorts, the main venue area of the Winter Olympic Games, and the Thaiwoo resort (Figure 2c). The vegetation loss caused by resorts was mainly due to the construction of ski trails and supporting facilities (such as hotels and the Winter Olympic Village). The most remarkable change occurred in the middle of the core area, which was obviously related to the construction of facilities in the Chongli competition zone, such as venues, the recently built high-speed railway station (Taizicheng Station), and the Yanchong Motorway (from Yanqing, Beijing to Chongli, Zhangjiakou). In the whole core area, the decrease in vegetation cover accounted for 52.3% (77.5%), of which the largest proportion was a relatively weak reduction, as can be seen from the large white area in Figure 2a,b.

### 3.2. The Vegetation Coverage Changes in the Chongli District of Zhangjiakou

**Figure 3:**
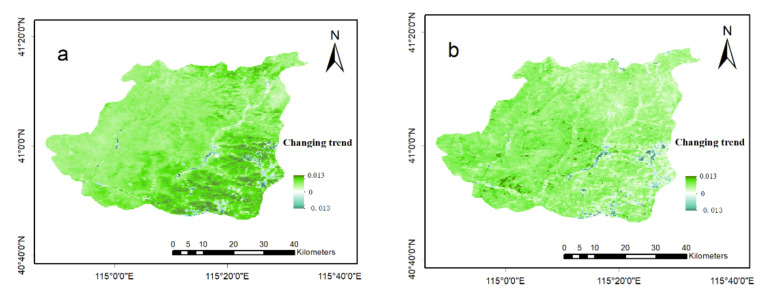
Increasing trend of vegetation of Chongli District, Zhangjiakou area, Hebei Province, China. (**a**,**b**) show the trend of vegetation change from 2000 to 2020 of Normalized Difference Vegetation Index (NDVI) and Enhanced Vegetation Index (EVI), respectively. The deeper the green, the greater the increase of vegetation coverage, and the opposite for blue.

The average NDVI value in Chongli District has been 0.72 (0.48) over the past 21 years, as seen in Figure 3. The vegetation increased significantly, with a θ value of 0.0084 (0.0064) and *p* < 0.001 (0.001). A total of 61.5% (60.8%) of the whole area showed an increasing trend, whereas the area where reduction occurred accounted for 38.5% (39.2%). This reduction was slight, mainly within the range of less than −0.0001 (−0.0001). The areas where reduction occurred were mainly concentrated in residential areas along the main traffic line.

### 3.3. The vegetation Coverage Variations in the Zhangjiakou Area

**Figure 4:**
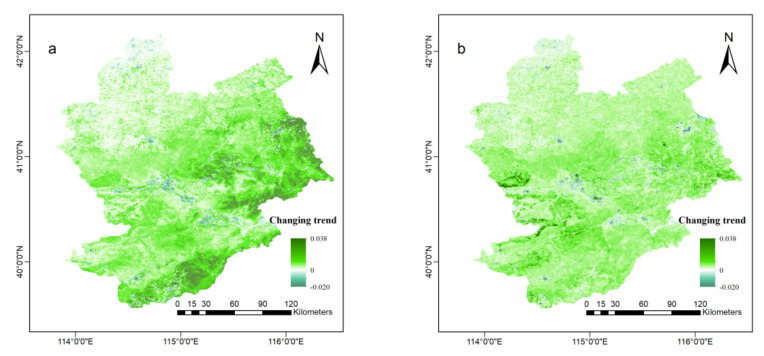
Increasing trend of vegetation of Zhangjiakou area, Hebei Province, China. (**a**,**b**) show the trend of vegetation change from 2000 to 2020 of Normalized Difference Vegetation Index (NDVI) and Enhanced Vegetation Index (EVI), respectively. The deeper the green, the greater the increase of vegetation coverage, and the opposite for blue.

The average NDVI value of the whole area in Zhangjiakou has been 0.64 (0.43) over the past 21 years. As shown in Figure 4, the vegetation increased significantly, with a θ value of 0.0071 (0.0057) and *p* < 0.001 (0.001). About 95.7% (94.2%) of the whole area showed an increasing trend, and this increase was mainly within the range of 0~0.010 (0.008). The increased areas were mainly concentrated in natural landscape areas with low populations. The areas where reduction occurred were mainly concentrated in urban areas with flat terrain and large populations.

## 4. Discussion

The large-scale construction of the Olympic Games usually brings about a series of environmental problems. Our studies, based on the vegetation coverage data, indicate that although the construction of the Beijing Winter Olympic Games has inevitably caused disturbance to vegetation coverage in the core area of the Zhangjiakou competition zone, the impact is limited and compensable. Our studies indicated that the vegetation coverage in the Zhangjiakou competition zone increased in half of the area (which was related to the afforestation of the surrounding mountains for landscape construction), while there was only a slight reduction for a large part of the decreased area. Moreover, the core area only accounts for a small partition of the Chongli District as a whole. Song et al. (2018) [19] reported that the total loss of area of natural ecological systems (forests, shrubs, and meadows) for a new ski resort for the 2022 Winter Olympic Games is 1.1727 km^2^, compared to the increase in vegetation coverage brought about by sustainable vegetation construction in the Zhangjiakou competition zone. The land-use change will lead to limited ecosystem function losses such as water and soil conservation, even in the core area. The limited plant damage during construction in the core area did not hinder the remarkable increase in vegetation coverage in Chongli District. For larger regions, such as the Zhangjiakou zone, these disturbances were even more negligible.

Since the 1990s, with the increasing awareness of the public to protect the environment, the International Olympic Committee has raised environmental protection and sustainable development to a strategic level, and the Olympic Organizing Committees of the host countries have paid more and more attention to environmental protection and sustainable development [20]. The 1994 Lillehammer Winter Olympic Games in Norway opened a new era of the Winter Olympic Games, integrating environmental protection into the special plans, actions, and the process of the organizing committee as a whole, becoming the first “green” Olympic Games [21]. The 2002 Salt Lake City Winter Olympics were the first Games after the International Olympic Organizing Committee officially established sports, culture, and environment as the three pillars of the Olympic movement, and established four environmental objectives [22]. In 2010, the Vancouver Organizing Committee for the Olympic Games specially set up a sustainability and resource management committee, taking environmental sustainability as one of the core strategies, and established a new global standard in the environmental protection of sports events. The venue of the 2014 Sochi Winter Olympic Games was located in a world heritage site, which elicited strong criticism from international media and environmental experts. To respond to these criticisms, the Sochi organizing committee established a green building standard for large-scale projects in Russia, and implemented many projects in environmental pollution control, animal and plant protection, and carbon emission reduction [22]. Pyeongchang, South Korea, has put forward the plan of a “green dream” since its bid. However, more than 58,000 trees, including some 500-year-old ancient trees and rare species, were destroyed to make way for the venue of the 2018 PyeongChang Winter Olympics [9]. Environmental activists said that it was an ecological disaster.

Unlike the Pyeongchang Winter Olympic competition area, the vegetation around the main competition zones of the Beijing Winter Olympic Games is mostly comprised of artificial forests newly built in the last 40 years, and there are almost no original trees or old trees. Despite this, synchronized with the construction of the 2022 Beijing Winter Olympic venues, a series of environmental protection measures have been implemented, including the transplanting of tens of thousands of trees from the mountains to a local botanical garden. The botanical garden has a planned area of around 100 hectares, mainly used to transplant trees and shrubs from the competition zones. To conserve native flora, more than 20,000 plants in the disturbed area, including the protected tree species *syringa reticulata*, *juglans mandshurica*, and *fraxinus rhynchophylla*, have been transplanted from the mountain areas to the local botanical garden as of yet. It is a big challenge to transplant plants that are growing on the mountains, particularly in the growing season when their roots are vulnerable to damage and dehydration. Forest experts have carried out an ecological survey on the disturbed area and conducted multiple rounds of trials and tests. Finally, they made a feasible transplanting plan. For example, to ensure the survival of transplanted trees, the gardeners increased the root excavation area, wrapped the trunks, and trimmed the crowns according to the species and volume of each tree to minimize their damage. During transplantation, rooting powders and nutrient solutions were applied. After these measures, the survival rate of transplantation was around 90%. In addition, in order to improve the corridor landscape and vegetation coverage in and around the competition area, the ecological restoration in local areas generally adopted large tree afforestation. Although the cost was high, this measure skipped the slow growth period of saplings and could quickly realize greening effects as well as landscape effects.

The Beijing Organizing Committee for the 2022 Winter Olympic Games has released its official “Beijing 2022 Olympic and Paralympic Winter Games Sustainability Plan”. The plan covers three key themes of “positive environmental impact”, “new development for the region”, and “better life for the people”. These 3 themes are supported by 12 actions, 37 key tasks, and 119 specific measures, including environmental protection in the competition zones, regional development, and the improvement of well-being for the host communities in Beijing and Zhangjiakou. For example, in the core area, wind power will account for a large proportion of the total energy; the use of electric heating and trams will greatly reduce carbon dioxide emissions.

All this helps the construction of the Beijing Winter Olympic Games to bring welfare, rather than disaster, to the local ecological environment. The vegetation coverage around the Zhangjiakou competition zone was increasingly enhanced from 2000 to 2020, even in the core area of the venue construction zone. Based on this, we have reason to believe that the Beijing Winter Olympic Games will become a green and environmentally friendly Olympic Games, and this grand event will be a typical positive case for the balancing of conservation and development of Winter Olympic projects, as well as for other mega-construction projects globally.

## 5. Conclusions

According to the remote sensing data from 2000 to 2020, the changes of vegetation coverage in the Zhangjiakou competition zone of the 2022 Beijing Winter Olympic Games were evaluated in this study. Our results showed that vegetation coverage around the Zhangjiakou competition zone was increasingly enhanced, even in the core area of the Winter Olympic Games construction zone. It indicated that the construction of venues, roads, and other facilities for the Beijing 2022 Winter Olympic Games led to land-use change, but the impact on vegetation coverage is negligible due to the intensive reforestation activities since the year that Beijing won the race to host the Winter Olympics. We conclude that the construction of tracks, traffic systems, and venues in mountainous areas for the Winter Olympic Games will inevitably cause disturbance to vegetation coverage, but as long as we take positive environmental compensation measures, such as large-scale afforestation, the Winter Olympic Games can bring more “green” and development, rather than environmental disaster.

## Figures and Tables

**Table 1:** Information on districts and counties of Zhangjiakou.

Districts or Counties	Area (km^2^)	Mean Altitude (m)	Abbreviation
Kangbao	3341	1422	KB
Guyuan	3589	1476	GY
Zhangbei	4181	1447	ZB
Shangyi	2650	1362	SY
Chicheng	5265	1209	CC
Chongli	2361	1490	CL
Wanquan	1120	1016	WQ
Qiaoxi Qiaodong	422	806	QQ
Xuanhua	2336	975	XH
Huaian	1683	1094	HA
High-Tech Zone	120	1145	HTZ
Huailai	1789	801	HL
Xiahuayuan	61	645	XHY
Zhuolu	2766	1117	ZL
Yangquan	1835	1062	YQ
Weixian	3196	1305	WX

## Data Availability

Publicly available datasets were analyzed in this study. These data can be found here: the NASA website (https://ladsweb.nascom.nasa.gov/search/ (accessed on 10 October 2020)); United States Geological Survey (USGS) website (https://earthexplorer.usgs.gov/ accessed on 10 October 2020)).
